# Congenital arhinia: A rare case

**DOI:** 10.4103/0971-9261.44771

**Published:** 2008

**Authors:** Abhishek Goyal, Vikesh Agrawal, V. K. Raina, D. Sharma

**Affiliations:** Pediatric Surgery Unit and Department of Surgery, NSCB Government Medical College, Jabalpur, Madhya Pradesh, India

**Keywords:** Arhinia, congenital malformations, nose

## Abstract

Congenital arhinia or absence of nose is a rare condition with only 30 cases reported so far. We report a rare case and briefly review the literature.

## INTRODUCTION

Congenital arhinia is an extremely rare condition. We report a case and briefly review the literature with respect to diagnosis and management.

## CASE REPORT

**Figure 1:**
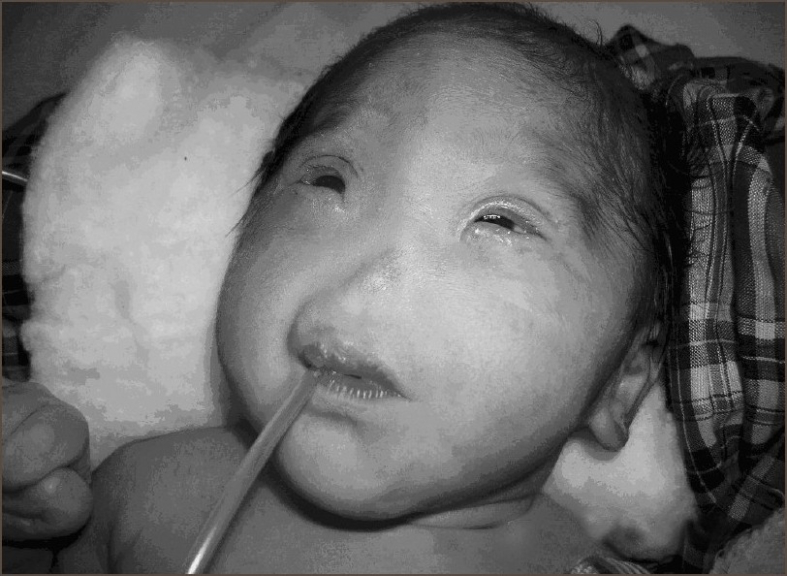
Clinical photograph

**Figure 2:**
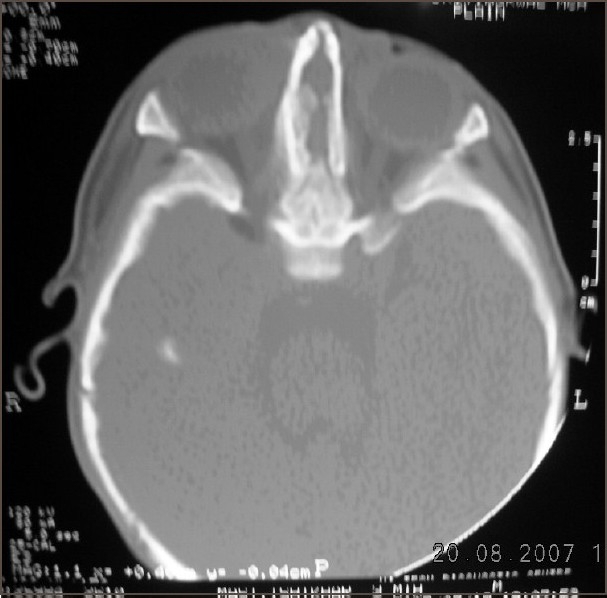
CT scan showing narrow nasal cavity with pyriform aperture and choanal atresia

A full-term neonate presented with respiratory distress and absence of external nose. He weighed 2.5 kg. There was clinical evidence of bony fusion at the site of nasal aperture, hypertelorism, and micropthalmia [Figure 1]. No other syndromic features were seen on routine systemic clinical examination. The chest radiograph and two-dimensional echocardiography were normal. Chromosome analysis was not done. Computed tomography (CT) of nasal region showed absence of nasal septum, bilateral choanal stenosis, microcephaly, and craniosynostosis. Brain window revealed no hydrocephalus and no obvious brain anomaly [Figure 2].

Initial stabilization was provided in the form of orogastric aspiration, maintenance of oral airway, and oxygenation. Simple oral airway was used which would allow passage of a No. 10 infant feeding tube also. Under general anesthesia and endotracheal intubation, emergency blind puncture of nasal diaphragm with bone trephine was done. A 14-Fr Ryle's tube was used for stenting so that posterior end lay in the nasopharynx and anterior end opened outside. This stent allowed suction and oxygenation. The stent was removed after 12 days and regular calibration was continued. But gradual restenosis occurred after one month. Eventually the infant learned oral breathing and spoon feeding; and is thriving.

## DISCUSSION

Congenital arhinia is defined as the absence of external nose, nasal cavities, and olfactory apparatus. It is an extremely rare condition with only 30 cases reported in literature so far.[1,2] It poses a challenge for pediatric, craniofacial, and plastic surgeons. It is classified as type I congenital nasal abnormality according to Losee's classification.[3] Bosma described arhinia hypertelorism micropthalmia syndrome associated with palatal abnormalities, deficient taste and smell, inguinal hernias, hypogonadotropic hypogonadism with cryptorchidism, and normal intelligence as associated malformations.[4,5]

Embryologically, formation of nose and bony canal begins in the third week and ends in the eighth week of intrauterine life. Arhinia results due to failed fusion of maxillary process and lateral nasal process. Associated cribriform plate fusion abnormalities result in olfactory agenesis.[1,3] Chromosomal aberrations like inversion and trisomy 9 have been reported in some cases of arhinia.[6]

CT scan determines the thickness of the atretic plate, choanal atresia, brain anomalies, and extent of micropthalmia and is an important tool in planning of surgical correction. MRI of brain is useful to delineate brain anomalies.[1,2]

The treatment of arhinia after birth typically involves treatment of airway obstruction and feeding difficulties. This aspect of management is of extreme importance to pediatric surgeons. Since neonates are obligate nasal breathers, simultaneous sucking and breathing requirement in neonates with arhinia leads to respiratory distress. Inspiration and expiration through the oral passage alone may result in thoracic retraction, thereby further exacerbating respiratory distress. Temporary measures like oral airway and orogastric feeding are successful. Canalization of nasal passage or tracheostomy may be required depending on severity of neonatal respiratory distress.[1,2,7]

Surgical reconstruction is very demanding and needs a team of pediatric, neuro, craniofacial, and plastic surgeons.

The components of repair include creation of orifice and reconstruction of external nose. Anterior aperture can be created by external approach and posterior orifice may be formed with endoscopic guidance.[2,3]

Reconstruction of external nose should be done near adolescence because growth of the reconstructed nose is unpredictable if done earlier. But the psychological trauma to the parents and the child may demand early correction which can be done earliest by the age of 4–5 years. It includes reconstruction of bony and cartilage framework along with mucosal lining and skin coverage of external nose.[1,7] Cartilage framework from costal cartilage placement in forehead and transfer of forehead flap with grafted framework for nose reconstruction is the most preferred option. Vertical distraction osteogenesis represents a modality for elongation of the mid face.[2,6]

Treatment of craniosynostosis and hypertelorism is done around 1-year of age before the brain growth is hampered. The procedures involved are various types of advancement cranioplasty.[2]

Antenatal ultrasound, preferably three dimensional with facial profile, can detect the condition earliest by 12–16 weeks of gestational age. Prenatal diagnosis has been reported in only one case during the second trimester of pregnancy.[5] Prognosis of such children is poor in terms of mental and physical development as well as cosmetic and functional outcomes. Antenatal diagnosis at an appropriate gestational age can provide the option of termination of pregnancy to the parents, especially if associated with other anomalies and trisomy.

Congenital arhinia is a rare anomaly and management is difficult. This case highlights the complexity of the condition with respect to surgical management. A multidisciplinary approach is essential along with expert neonatal medical and surgical care for the management.
